# Quo vadis, gemeinsames Notfallleitsystem? Standardisierte Notrufabfrage in der Berliner Leitstelle

**DOI:** 10.1007/s10049-022-01073-1

**Published:** 2022-09-07

**Authors:** Florian Breuer, Paul Brettschneider, Stefan Poloczek, Christopher Pommerenke, Justus Wolff, Janosch Dahmen

**Affiliations:** 1 Berliner Feuerwehr Einsatzvorbereitung Rettungsdienst, Berlin, Deutschland; 2 Ärztliche Leitung Rettungsdienst Berliner Feuerwehr, Berlin, Deutschland; 3 Ärztliche Leitung Rettungsdienst Rheinisch-Bergischer Kreis, Amt für Feuerschutz und Rettungswesen, Am Rübezahlwald 7, 51469 Bergisch Gladbach, Deutschland; 4 Referat Leitstelle, Berliner Feuerwehr, Berlin, Deutschland; 5 Universität Witten/Herdecke, Fakultät für Gesundheit, Department Humanmedizin, Witten/Herdecke, Deutschland; 6 Charité – Universitätsmedizin Berlin, Berlin, Deutschland

**Keywords:** Präklinische Versorgung, Rettungsdienst, Notfallmedizin, Qualitätsmanagement, Ärztlicher Leiter Rettungsdienst, Preclinical treatment, Emergency medical services, Emergency medicine, Quality assurance, EMS medical director

## Abstract

Die Zahl von Hilfeersuchen an die Notrufnummer 112 sowie die Einsatzzahlen im Rettungsdienst nehmen seit vielen Jahren insbesondere in urbanen Regionen Deutschlands massiv zu. Die Leitstelle kann hier als Steuerungsinstrument mit Lotsenfunktion genutzt werden, um neue Wege bei der Notrufbearbeitung sowie der Disposition von Einsatzmitteln zu gehen und das Anrufaufkommen hiermit möglichst effizient zu leiten. Es werden die standardisierte Notrufabfrage sowie weitere wichtige Strukturen und Pfade anhand der Leitstelle der Berliner Feuerwehr mit Fokus auf medizinische Notrufe, auch vor dem Hintergrund der COVID-19-Pandemie, dargelegt und näher erläutert. Strukturierte und standardisierte Notrufabfrage sind voneinander abzugrenzen, wobei die standardisierte Notrufabfrage verbindlich und auf Grundlage internationaler Standards mit hoher Reliabilität erfolgt. Ein strukturiertes Qualitätsmanagement sorgt für eine regeltreue Anwendung des Protokolls. Durch eine verbesserte Zusammenarbeit und eine elektronische Schnittstelle zur Leitstelle der kassenärztlichen Vereinigung können niedrigprioritäre Einsätze dorthin weitergeleitet werden. Interprofessionelle Teams im Rettungsdienst können zielgerichtet die Versorgung bestimmter Patientengruppen verbessern sowie zur Vermeidung von Transporten beitragen. Die standardisierte, softwaregestützte Notrufabfrage entspricht dem aktuellen Stand der Wissenschaft, wobei eine flächendeckende Einführung sinnvoll erscheint. Des Weiteren empfehlen sich eine intensive Zusammenarbeit von Leitstellen der Notfallrettung und der kassenärztlichen Vereinigung, die bedarfsadaptierte Einführung spezialisierter Einsatzmittel sowie die applikationsunterstützte Alarmierung von Ersthelfern.

## Abstract (en)

The call volume in emergency medical service (EMS) dispatch centers has seen a drastic increase for many years now, especially looking at urban regions of Germany. In this context, the control mechanisms of the EMS dispatch center can be utilized to break new ground regarding the handling of emergency calls and dispatch practice in order to manage incoming calls as efficiently as possible. This article clearly explains standardized protocol-based emergency medical call taking, internal structuring of control centers and pathways*, *also during the COVID-19 pandemic, using the Berlin EMS dispatch *c*enter as an example. The terms structured and standardized protocol-based emergency medical call taking should be differentiated, whereby the standardized call taking process is more binding and based on international standards with high reliability. Quality management measures ensure that the protocol is applied in accordance with the regulations. Improved collaboration and automated transfer of data between EMS dispatch centers and the control centers for non-life-threatening physician on-call services enable low-priority calls to be forwarded on a regular basis. Interprofessional teams in EMS can improve the care of specific patient groups in a targeted manner and avoid transport to emergency departments. Standardized protocol-based and software-based emergency call taking currently represents best practice according to medical science, supporting a nationwide implementation. Furthermore, an intensive collaboration between EMS control centers and control centers for non-life-threatening physician on-call services is recommended as well as the introduction of specialized EMS resources and app-based alerting of first responders.

## Hintergrund

Die Einsatzzahlen im Rettungsdienst nehmen seit vielen Jahren insbesondere in urbanen Regionen in Deutschland und den europäischen Nachbarländern stetig zu. Als ursächliche Einflussfaktoren werden mitunter eine zunehmende Verstädterung, Vereinsamung und der demografische Wandel einhergehend mit einer veränderten Mobilität und Morbidität der Bevölkerung diskutiert. [31]. Ungeachtet dessen ist aber auch davon auszugehen, dass ein verändertes Gesundheitsbewusstsein der Bevölkerung, unterschiedliche sozioökonomische Einflussfaktoren, aber auch das Fehlen von Alternativen in der Gesundheitsversorgung und anderen Hilfeleistungsangeboten der Sozialsysteme eine entscheidende Rolle spielen [1]. Im Ergebnis einer repräsentativen Bevölkerungsumfrage im Land Berlin konnte geschlussfolgert werden, dass insbesondere die fehlende Verfügbarkeit von zuverlässigen Alternativen, die seitens der Bevölkerung anstelle des Notrufs 112 in Anspruch genommen werden könnten, zur vermehrten Nutzung beiträgt [10]. In dem Zusammenhang gibt es Hinweise, dass sogenannte Frequent User in bedeutendem Umfang wiederholt den Notruf beanspruchen und damit einen zahlenmäßig relevanten Einsatzanteil in Leitstellen generieren [3, 4]. Einzelne Arbeiten lassen vermuten, dass sogenannte niedrigprioritäre Hilfeersuchen zunehmen oder zumindest konstant einen hohen Anteil des Notrufaufkommens ausmachen. So lag in der Stadt Köln der Anteil an nicht akut lebensbedrohlichen Einsätzen in einem Zeitraum von einem Jahr konstant bei 60 % [19]. Der Notrufnummer 112 bzw. der Leitstelle als zentrale Steuerungs- und Lotsenfunktion kommt zunehmend eine ganz entscheidende Bedeutung zu, die auch die Möglichkeit erfordert, im Ergebnis der Notrufabfrage alternative Versorgungsformen oder differenziert innovative Einsatzmittel entsprechend der spezifischen Hilfsbedürftigkeit zu entsenden [24].

In der Notfallmedizin sind Standards in Form von Standard Operating Procedures (SOP) längst nicht mehr wegzudenken. Auch im Rahmen der technischen Hilfeleistung und im Brandschutz sind Regelungen zum Einsatzablauf bei den Feuerwehren in sogenannten Standardeinsatzregeln (SER) niedergeschrieben. Es entspricht nicht mehr dem aktuellen Stand von Wissenschaft und Technik, wenn in Leitstellen der nichtpolizeilichen Gefahrenabwehr teilweise noch eine freie Abfrage oder allenfalls eine strukturierte Abfrage anhand von Leitfragen erfolgt, die lediglich unverbindlich zur Orientierung dienen.

In einer Umfrage zur Etablierung von strukturierten und standardisierten Notrufabfragesystemen konnte gezeigt werden, dass die Einführung einer strukturierten oder standardisierten Notrufabfrage in Leitstellen positive Effekte auf Abfragequalität, Dispositionsqualität und Anleitung zur Ersten Hilfe hatte. Dennoch nutzte nur die Hälfte der befragten Leitstellen ein entsprechendes Verfahren [23]. Auch internationale Arbeiten zeigen die Vorteile einer standardisierten Notrufabfrage [34].

Teilweise wird die standardisierte Notrufabfrage nicht hinreichend von einer strukturierten Notrufabfrage abgegrenzt. Wesentliche Unterscheidungsmerkmale zeigt Tab. 1. Unter Anwendung einer standardisierten Notrufabfrage erfolgt die Notrufabfrage verbindlich softwaregeführt auf der Grundlage internationaler Standards. Diese sind wissenschaftlich evaluiert und validiert, personenunabhängig und reproduzierbar.

**Table 1:** 

	Strukturierte Notrufabfrage	Standardisierte Notrufabfrage
Vorgegebene Gesprächsstruktur	Ja	Ja
Genauer Wortlaut der Fragen vorgegeben	Kann	Ja
Automatische Dokumentation der Abfrageergebnisse	Kann	Ja
Übergabe der Abfrageergebnisse an das Einsatzleitsystem	Kann	Ja
Generierung eines eindeutigen Codes	Nein	Ja
Fragen sind evaluierbar	Kann	Ja
Fragen evidenzbasiert	Nein	Soll
Qualitätsmanagement mittels Auswertung	Kann	Ja
Kontextabhängige Dynamisierung der Reihenfolge von Fragen	Nein	Soll
Erfüllt rechtliche Dokumentationserfordernisse	Kann	Ja
Speicherung aller Fragestellungen und Antworten mit Zeitstempel	Nein	Ja

Durch die Anwendung einer standardisierten Notrufabfrage ist es möglich, dass spezifische Protokolle zur Anleitung von ersten Hinweisen und Sofortmaßnahmen regelhaft und verlässlich zum Einsatz kommen und dieses auch zuverlässig rechtssicher dokumentiert wird.

Bei der Notrufabfrage ist zu beachten, dass es auch hier mehr und mehr zu haftungsrechtlichen Auseinandersetzungen mit Patientinnen und Patienten oder Angehörigen kommt. Im Fokus entsprechender gerichtlicher Auseinandersetzungen steht dabei einerseits die Organisationsverantwortung für die Umsetzung des aktuellen Stands der Wissenschaft – insbesondere medizinischer Leitlinien in der Leitstellenarbeit – und andererseits die individuelle Umsetzung entsprechender Empfehlungen durch das Personal der Leitstellen. Hierbei ist zu berücksichtigen, dass in Rechtsurteilen bereits festgestellt wurde, dass bei Fehlentscheidungen der Leitstelle, wie zum Beispiel einer falschen Disposition, die Rechtsgrundlagen der Arzthaftung Anwendung finden [27]. Dementsprechend ist die gesamte Dokumentation der Leitstelle, einschließlich des gesamten Notrufgesprächs, der Dispositionsentscheidungen und softwaregeführten Notrufabfrage, auch als Bestandteil der Behandlungsakte für einen Zeitraum von 10 Jahren (§ 630f BGB) aufzubewahren. Dies wurde in Berlin inzwischen in der Landesgesetzgebung für die Speicherfristen von Notrufgesprächen präzisiert, sodass nun eine Aufbewahrung für die Dauer von 10 Jahren erfolgt (§ 4 Absatz 2 RDG Berlin; [30]).

Die „Leitlinien zur Wiederbelebung“ des European Resuscitation Council (ERC) aus dem Jahr 2021 empfehlen die Etablierung einer Abfrageunterstützung zur Erkennung eines Herz-Kreislauf-Stillstands. Demnach sollen Leitstellen standardisierte Kriterien und Algorithmen einführen, um festzustellen, ob sich Patientinnen und Patienten zum Zeitpunkt des Notrufs in einem Kreislaufstillstand befinden. Weiterhin sollen Leitstellen über Systeme verfügen, welche sicherstellen, dass die Bearbeiterinnen und Bearbeiter von Notrufen in den Leitstellen die Anleitung zu Wiederbelebungsmaßnahmen für nicht reagierende und nicht normal atmende Personen in jedem Fall zuverlässig und vollständig geben. Derartige wissenschaftliche Fachempfehlungen in Leitlinien sind nur auf der Basis von standardisierten Notrufabfragesystemen verlässlich umsetzbar und rechtssicher überprüfbar [29].

Weiterhin ist eine standardisierte Notrufabfrage auch eine wesentliche Voraussetzung für die differenzierte Entsendung von Rettungsmitteln. Hierbei ist insbesondere der effiziente Einsatz von Notfallsanitätern zu nennen, die befähigt werden, heilkundliche Maßnahmen auf dem Kompetenzniveau „Beherrschen“ durchzuführen. Darüber hinaus erfordert auch die sinnvolle Anbindung von besonders erfahrenen Notärzten auf dem Kompetenzniveau „Experte“ zur vollumfänglichen Sicherstellung des Facharztstandards im Rettungsdienst, beispielsweise in Form des Oberarztes vom Dienst (OAvD), die Anbindung mittels standardisierter Notrufabfrage [7, 9].

Nach Auffassung der Autoren tragen Standards dazu bei, Fehler zu vermeiden oder zu reduzieren, wodurch letztendlich die Patientensicherheit erhöht und die Rechtssicherheit gesteigert wird. Die standardisierte Notrufabfrage stellt außerdem die Grundlage für eine sinnvolle Umsetzung von Ersthelfersystemen, die rechtssichere Disposition insbesondere nach der „Nächstes-Fahrzeug-Strategie“, sowie die Etablierung eines Telenotarztsystems dar. Weiterhin ermöglicht die standardisierte Notrufabfrage auch eine zielgerichtete Bearbeitung von niedrigprioritäten Hilfeersuchen mit Möglichkeit der Weiterleitung an andere Versorgungseinrichtungen.

## Standardisierte Notrufabfrage in Berlin

In der Leitstelle der Berliner Feuerwehr gehen täglich durchschnittlich 2300 Notrufe ein. Zu durchschnittlich 1285 Einsätzen (davon 1164 Einsätze mit Fokus auf die medizinische Gefahrenabwehr und 121 Einsätze mit Fokus auf die technische Hilfeleistung bzw. den Brandschutz) werden Einsatzmittel entsandt [2]. Für die Notrufabfrage sind zeitgleich in der Regel 13 Abfrageplätze besetzt.

Im Rettungsdienstgesetz für das Land Berlin (RDG) ist im § 8 Abs. 1 geregelt, dass Notrufe, die unter der Notrufnummer 112 eingehen, von der integrierten Leitstelle der Berliner Feuerwehr regelmäßig unter Anwendung einer standardisierten Notrufabfrage beantwortet werden. Weiterhin ist hier auch beschrieben, dass die standardisierte Notrufabfrage die telefonische Anleitung zu Erste-Hilfe-Maßnahmen beinhaltet und die integrierte Leitstelle das auf der Basis der Grundlage der standardisierten Notrufabfrage ermittelte und für den Einsatz geeignete, nächstgelegene Fahrzeugaufgebot entsendet [30].

Die Notrufabfrage bei der Berliner Feuerwehr erfolgt unter Anwendung der Protokolle des Medical Priority Dispatch System (MPDS) sowie des Fire Priority Dispatch System (FPDS; [16]). Je nach geschildertem Hilfeersuchen bzw. geschilderter Schadenslage wählen die aufnehmenden Call Taker (Notrufsachbearbeiterinnen und Notrufsachbearbeiter) die passende Disziplin für die standardisierte Notrufabfrage aus. Die Protokolle leiten die Call Taker durch einen standardisierten Prozess. Hierbei wird der Patientenzustand anhand objektiver Kriterien im Protokoll erfasst. Je nach Beschwerdebild und Verletzungsmuster werden spezielle Fragen gestellt und vordefinierte Antworten erfasst. Der Aufbau und der Inhalt der Protokolle sind international in allen beteiligten Leitstellen verbindlich und können durch die Berliner Feuerwehr nur an sehr wenigen und streng definierten Stellen eigenständig regional modifiziert werden. Änderungen des Protokolls erfolgen stattdessen in der Regel einheitlich im Rahmen regelmäßiger Reviews im wissenschaftlichen Board von Priority Dispatch Systems. Ein entsprechendes Meldebild führt somit immer zum gleichen Einsatzstichwort und in der Folge auch zum gleichen Dispositionsergebnis (Infobox 1).

Anhand der standardisierten Codes ist dementsprechend eine situative Alarmierung von Einsatzmitteln durch die Anbindung an die Alarm- und Ausrückeordnung vordefiniert. Hier ist auch eine Abgabe an Dritte möglich, sofern sich keine primäre Zuständigkeit für den Rettungsdienst ergibt. Weiterhin wird anhand des Abfrageergebnisses festgestellt, in welcher Beschaffenheit erste Hinweise und Anweisungen zu lebensrettenden Sofortmaßnahmen telefonisch gegeben werden müssen. Hierbei werden die Anrufer bis zum Eintreffen der Einsatzkräfte durch die Call Taker angeleitet. Die standardisierte Notrufabfrage schließt mit der obligaten Anweisung *„Wenn sich der Zustand verschlechtert […], rufen Sie uns sofort wieder an, damit wir Ihnen weiterhelfen können“.*

Neben der Notrufabfrage in deutscher Sprache wird das Protokoll in Berlin auch englischsprachig regelhaft angewendet. Hierbei können sowohl die Fragen und Anweisungen der Leitstelle als auch die Antwortoptionen der Anrufer mit einem Mausklick ins Englische übersetzt werden. Dies stellt die Bearbeitung anhand des geforderten Standards sicher. Weitere Sprachen werden durch das Protokoll ebenfalls abgebildet.

Die standardisierte Notrufabfrage ermöglicht darüber hinaus auch kurzfristige Reaktionen, beispielsweise auf aktuelle pandemische Lagen. So konnten im Rahmen der COVID-19-Pandemie durch die Anwendung eines gesonderten Protokolls „Pandemie/Epidemie/Ausbruch“ mögliche COVID-19-Erkrankungen sowie die Auswirkungen auf das rettungsdienstliche Einsatzgeschehen frühzeitig erkannt werden. Durch Hinzufügen eines Zusatzes ARE (akute respiratorische Erkrankung) zum Einsatzstichwort werden die Einsatzkräfte bereits mit der Alarmierung sensibilisiert [5, 6].

## Anbindung von Einsatzstichworten – Code Reviews

Im Ergebnis der Notrufabfrage wird somit in Abhängigkeit des ermittelten Leitsymptoms oder der geschilderten Beschwerden aktuell einer von 5564 Dispatch Codes generiert, wobei 2139 Dispatch Codes aus dem MPDS-Protokoll und 3425 Dispatch Codes aus dem FPDS-Protokoll hervorgehen. Diese Codes sind dynamisch, da sich durch Anpassungen an aktuelle Leitlinie und Empfehlungen und Softwareaktualisierungen fortlaufend Veränderungen ergeben, um hinreichend dem aktuellen Stand der Wissenschaft und Rechtsprechung zu genügen.

An diese Codes wiederum erfolgt die Anbindung der Alarm- und Ausrückeordnung mit entsprechenden modular aufgebauten Einsatzstichwörtern. Somit erfolgt auf dieser Ebene die Anbindung von Einsatzmitteln unter Berücksichtigung von Dringlichkeit, aber auch von notwendiger Qualifikation oder Ausstattung. Ein wesentlicher Vorteil hierbei ist die Möglichkeit einer abgestuften Reaktion. Die Anbindung von Einsatzaufgeboten (Qualifikation und Ausstattung) erfolgt dementsprechend bedarfsabhängig, wobei es hier zum einen Codes mit einer hohen Dringlichkeit gibt (Delta, Echo), genauso aber weniger dringliche Codes (Alpha, Bravo, Charlie) bis hin zu denjenigen, an die beispielsweise die Abgabe an die kassenärztliche Vereinigung (KV) angebunden ist (Omega; Tab. 2).

**Table 2:** 

Dringlichkeitsstufen	Beispiel
E = Echo	Feststellung höchster Dringlichkeit – Alarmierung wird unverzüglich in Gang gesetzt (z. B. Reanimation)
D = Delta	RTW und NEF (z. B. Brustschmerz mit Kaltschweißigkeit)
C = Charlie	RTW (z. B. Brustschmerz mit normaler Atmung ohne Kaltschweißigkeit, ohne Herzinfarkt‑/Angina-pectoris-Vorgeschichte)
B = Bravo	RTW (z. B. schwere Augenverletzung)
A = Alpha	KTW Typ B (z. B. Bauchschmerzen ohne Bewusstseinstrübung oder sonstige Risikofaktoren)
Ω = Omega	Für geplante Weiterleitung (z. B. an den kassenärztlichen Bereitschaftsdienst)

*KTW* Krankentransportwagen, *NEF* Notarzteinsatzfahrzeug, *RTW* Rettungswagen

Die Zuständigkeit der Ärztlichen Leitung Rettungsdienst für die Festlegung der Strategien zur Bearbeitung von medizinischen Hilfeersuchen durch die Leitstelle sowie die Festlegung der Strategie der Disposition rettungsdienstlicher Einsatzmittel durch die Leitstelle geht bereits aus der Empfehlung der Bundesärztekammer im Jahr 2013 hervor [8]. In Berlin wurden diese Verantwortlichkeiten zusätzlich im Rettungsdienstgesetz verbindlich verankert [30]. Zur Qualitätssicherung finden dementsprechend mindestens quartalsweise sogenannte Code Reviews unter Beteiligung der Ärztlichen Leitung Rettungsdienst und aller sonstigen Prozessverantwortlichen statt, in denen die Codeanbindung einzelner Stichwörter systematisch evaluiert und ggf. angepasst wird. Auf der Basis einer digitalen Einsatzdokumentation sowie der Daten aus dem Einsatzleitsystem ist es inzwischen beispielsweise auch möglich, Codes aus der Notrufabfrage retrospektiv mit dem im Einsatzprotokoll dokumentierten mNACA-Score oder anderen im Minimalen Notfalldatensatz (MIND) dokumentierten Parametern in Beziehung zu setzen. Weiterhin werden anlassbezogen auch Einsatzprotokolle qualitativ ausgewertet, um zu bewerten, welche Art von Einsätzen und welche Verdachtsdiagnosen sich hinter den Alarmierungen zu bestimmten Einsatzcodes verbergen. Auf diese Weise soll einer Untertriage, aber auch einer Übertriage bestmöglich entgegengewirkt werden.

## Qualitätsmanagement

Anforderungen an die Qualität in der standardisierten Notrufabfrage können nur mit einem ganzheitlichen Ansatz geplant, gestaltet und überprüft werden. Ein systematisches Qualitätsmanagement ist demnach erforderlich, um die Qualität kontinuierlich zu verbessern und zu sichern.

Eine mangelhafte Compliance in den Protokollanwendungen wirkt sich folglich auf die Dispositionsqualität und die letztlich auch auf die unmittelbare Patientenversorgung aus [17]. Daher geht die Etablierung eines standardisierten Notrufabfrageprotokolls mit einem umfassenden Qualitätsmanagement zwingend einher.

### Qualitätssicherung

Die Sicherung der Qualität im Rettungsdienst bedeutet, notfallmedizinische Leistungen in unveränderter, gleichbleibender und definierter Qualität zu erbringen, wobei mit der Qualitätssicherung zunächst noch keine Qualitätsverbesserung verbunden ist. Vielmehr soll die Beschaffenheit der Leistung erhalten bleiben und langfristig gesichert werden.

Bei der standardisierten Notrufbearbeitung liegt der Fokus hier in der objektiven Leistungsmessung der Notrufbearbeitung anhand von regelmäßigen QM-Fallauswertungen. Bei den Fallprüfungen handelt es sich um stattgefundene Notrufgespräche, die nach einem Zufallsprinzip ausgewählt und nach einem vorgebenden Leistungsstandard bewertet werden. Je nach Bewertung ergibt sich eine Befolgungsstufe und die daran gebotene Beschaffenheit des QM-Feedbacks.

Neben der Zufallsauswahl, die sich an einem festen Schlüssel orientiert, um die Qualität repräsentativ zu erfassen, werden auch gezielte QM-Fallauswertungen vorgenommen (Infobox 2). Diese unterliegen den gleichen Bewertungsstandards wie die der Zufallsauswertung. Sie dient der Klärung einer spezifischen Fragestellung oder einem eingegrenzten Themenbereich. Die Anwendung der Abfrageprotokolle MPDS und FPDS sieht eine erstmalige Zertifizierung und fortlaufend eine zweijährliche Rezertifizierung vor. Seitens der Call Taker ist innerhalb der 2 Jahre ein 36 h umfassendes Fort- und Weiterbildungsprogramm zu absolvieren. Dieses muss durch die Leitstelle individuell gestaltet werden und dient wesentlich der Qualitätsverbesserung.

### Qualitätsverbesserung

Rahmenbedingungen für Feedback sowie die Aus- und Weiterbildung tragen ganz wesentlich zu Qualitätsverbesserung bei.

Das Feedback schließt sich an die Leistungsmessung der zufälligen und/oder gezielten Fallauswertung an. Es soll nicht nur die Protokoll-Compliance, sondern gleichzeitig Entwicklungspotenzial aufzeigen, da neben der Interaktion mit den Meldenden kommunikative, arbeitsorganisatorische wie auch EDV-technische Fähigkeiten gefordert sind. Hierfür hat sich in Berlin der Ansatz eines Fallsimulationstrainings etabliert. Neben dem Leistungsfeedback werden hierbei auch Notrufe simuliert. Diese sind entsprechend vorbereitet und sollen den Call Takern spezielle Bearbeitungspfade im Protokoll und dafür notwendige Kommunikationstechniken aufzeigen.

Weitere Fortbildungsformate wie Präsenzschulungen, eLearning oder Seminare müssen ebenfalls angeboten werden, um den erforderlichen Fortbildungsumfang sicherzustellen.

Ein wirksames Modell stellt die QM-Supervision im operativen Leitstellenbetrieb dar. Die Funktion des Supervisors soll unterstützend in schwierige Gespräche eingreifen oder auch parallele Arbeiten im Sinne des Notrufgesprächs in die Wege leiten. Weiterhin aktiviert sich diese Rolle bei definierten Einsätzen wie bspw. einer Reanimation oder einem schweren Traumaeinsatz selbst, um die Notrufbearbeitung zu unterstützen. Ein Feedback und, wenn notwendig, ein individuelles Coaching, erfolgen somit „on the job“. Die QM-Supervision hat insbesondere bei der Einarbeitung neuer Mitarbeiter einen sehr hohen Stellenwert.

### Qualitätsmanagementsystem

**Figure 1:**
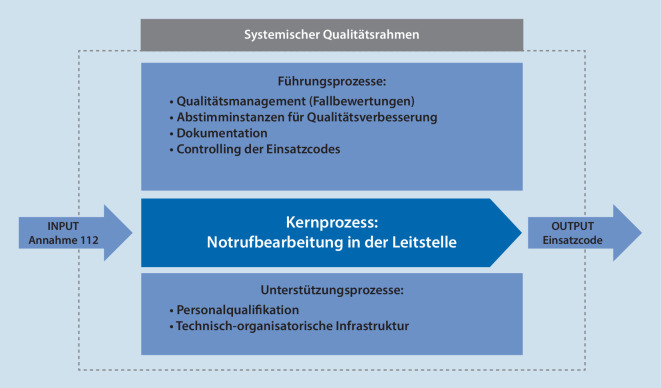

Alle Führungs- und Unterstützungsprozesse gilt es kontinuierlich an den Bedürfnissen des Kernprozesses der standardisierten Notrufbearbeitung auszurichten (Abb. 1). Die International Academy of Emergency Dispatch (IAED) hat hierzu einen Akkreditierungsleitfaden verfasst. Sofern alle Punkte vollumfänglich erfüllt werden, erfolgt die Akkreditierung als „Center of Excellence“. Dieses Ziel hat die Leitstelle der Berliner Feuerwehr noch nicht erreicht, sieht es weiterhin aber als sehr erstrebenswert an.

Ein wichtiger Fokus ergibt sich aus den dreigliedrigen Abstimminstanzen. Das QM-Team ist für die Auswertung und das leistungsfördernde Feedback verantwortlich, also unmittelbar für die Qualitätssicherung und die mitarbeiterbezogene Leistungssteigerung. Der QM-Ausschuss setzt sich aus verschiedenen Rollen der Leitstelle zusammen und evaluiert regelmäßig die Arbeit des QM-Teams. Er überprüft auch die Wirksamkeit und Effizienz der Führungs- und Unterstützungsprozesse und nimmt notwendige Datenanalysen und Interpretationen zu speziellen Themen vor. Der QM-Lenkungsausschuss ist das höchste Gremium, worin auch die Ärztliche Leitung Rettungsdienst vertreten ist. Hier werden gesamtorganisatorische Entscheidungen für die standardisierte Notrufbearbeitung getroffen.

Neben den Abstimminstanzen werden regelmäßig auch wichtige Stakeholder über die Arbeit und Funktion der standardisierten Notrufabfrage informiert und in die weitere Entwicklung einbezogen (z. B. Notärzte, Rettungsdienstfachpersonal, Auszubildende der Rettungsdienstschule, Führungskräfte der Feuerwachen, Vertreter der kassenärztlichen Vereinigung sowie Interessierte aus Forschung und Politik.

## Optimierung der Dispositionsstrategie

### „Nächstes-Fahrzeug-Strategie“ und softwaregestützte Austauschdisposition

Ein wesentliches Qualitätsmerkmal einer Leitstelle ist die sichere Identifikation von lebensbedrohlichen Hilfeersuchen und damit einhergehend die frühzeitige Zuordnung von denjenigen Ressourcen, die für den Einsatz erforderlich sind. Hierzu ist es erforderlich, dass die GPS-Standortdaten der Einsatzmittel für das Einsatzleitsystem verfügbar sind und unter Rückgriff auf diese ein Einsatzmittelvorschlag erfolgen kann. Auf diese Weise ist es möglich, dass insbesondere bei besonders zeitkritischen Hilfeersuchen, wie etwa einer gemeldeten Reanimationssituation, sichergestellt ist, dass nachweislich das nächstgelegene geeignete Rettungsmittel, beispielsweise im Sinne eines Voralarms, schnellstmöglich zur Einsatzstelle entsendet wird. Eine verlängerte Reaktionszeit führt nachweislich zu einer erhöhten Sterblichkeit, insbesondere bei Tracer-Diagnosen wie Herzinfarkt und Schlaganfall [36]. Durch eine Ortung der nächstgelegenen Fahrzeuge kann die Sterblichkeit gesenkt werden [37]. Es sollte darüber hinaus softwarebasiert im Einsatzleitsystem sichergestellt werden, dass insbesondere bei derartigen besonders zeitkritischen Notfällen über eine automatische, georeferenzierte Hintergrundüberwachung aller Einsatzmittel gewährleistet wird, dass im Falle einer bereits erfolgten Disposition und zwischenzeitlich günstigeren Verfügbarkeit eines anderen gleichwertigen Einsatzmittels mit besserer Eintreffzeitprognose dieses über eine automatische Austauschdisposition zum Einsatzort entsendet wird. Weiterhin müssen Standardarbeitsabläufe, die sich auch systemseitig abbilden lassen, existieren, die es ermöglichen, auf der Basis von Rückmeldungen die Alarmstufe schnellstmöglich zu erhöhen.

### Anbindung von First Respondern (FR2/FR7)

Die Hilfsfrist als planerische Größe hat nur bedingt Einfluss auf das therapiefreie Intervall, sodass es auch in einem großstädtischen Rettungsdienst sinnvoll und notwendig ist, ein First-Responder-System vorzuhalten [28]. Wesentliche Determinante für die effiziente Anbindung von First Respondern ist die differenzierte Anbindung an ausgewählte Einsatzcodes, weiterhin muss gewährleistet sein, dass der First Responder zeitlich vor den Einsatzkräften der Notfallrettung vor Ort eintrifft. Die standardisierte Notrufabfrage bietet die Möglichkeit, First Responder in Abhängigkeit von Dringlichkeit und medizinischer Komplexität anzubinden. Es konnte gezeigt werden, dass mittels Anwendung einer standardisierten Notrufabfrage der Einsatz von First Respondern verringert werden kann [35]. Dementsprechend werden in Berlin First Responder systematisch immer dann entsendet, wenn ein berechneter Zeitvorteil von 2 min (FR2) oder 7 min (FR7) vorliegt. Bei Reanimationen erfolgt ebenfalls die Entsendung eines First-Responder-Fahrzeugs bei jedem errechneten Zeitvorteil. Der Zusatz FR2 ist bei gemeldeter Bewusstlosigkeit bei Einsätzen mit Notarzt vorgesehen, bei der absehbar keine Reanimationssituation vorliegt. Der Zusatz FR7 erfolgt bei gemeldeten Notfallleitsymptomen (Bewusstseinstrübung, Brustschmerzen, Atembeschwerden, starke Blutung), da bei derartigen Meldungen mit einer schnellen Verschlechterung des Zustands gerechnet werden muss, wenn nicht baldmöglichst Erste Hilfe geleistet wird. Die Anbindung erfolgt sowohl bei Einsätzen mit als auch ohne Notarzt, wobei diese abhängig von der Dringlichkeit ist (Tab. 3). Die Codes werden im Rahmen der Code Reviews regelmäßig betrachtet.

**Table 3:** 

Stichwort mit Zusatz	Meldebild und EZPV	Sofortmaßnahmen durch die Leitstelle
NOTF. NA (REA. #EHA.)	Reanimationen, die nicht traumatisch bedingt sind und bei denen eine Gefährdung der Ersthelfer ausgeschlossen ist (EZPV: jeder)	Telefonreanimation, ggf. Einsatz des AED, wenn vorhanden
NOTF. NA (FR2.)	Alle Codes mit Notarztanbindung, bei denen eine Bewusstlosigkeit gemeldet ist (EZPV: 2 min)	Patient in Rückenlage verbringen und Atemwege überstrecken; ggf. Vorbereitung AED, wenn vorhanden
NOTF. NA (FR7.)	Alle Codes mit höherer Dringlichkeit (Delta) und Notarztanbindung, bei denen von einer akuten Vitalbedrohung ausgegangen werden muss (EZPV: 7 min)	Situativ; ggf. auch am Telefon bleiben, ggf. Vorbereitung AED, wenn vorhanden
NOTF. (FR7.)	Alle Codes, bei denen eine starke Blutung gemeldet ist (Bravo) (EZPV: 7 min)	Blutung stillen, ggf. Amputation versorgen

*AED* automatischer externer Defibrillator, *EZPV* Eintreffzeitprognosenvorteil

### Ersthelfer-App

Im Jahr 2020 wurde eine Ersthelfer-App eingeführt, die es ermöglicht, auf der Basis der standardisierten Notrufabfrage Ersthelferinnen und Ersthelfer zu aktivieren, um bei reanimationspflichtigen Patienten frühestmöglich mit der Wiederbelebung zu beginnen. Hierbei erfolgt systemseitig eine zweistufige Ortung: Im Ruhebetrieb wird der ungefähre Standort des Ersthelfers an das Einsatzleitsystem übermittelt und erst dann, wenn ein sogenannter Reanimationscode vorliegt, wird der exakte Standort geortet und übermittelt. Dann werden bis zu drei Ersthelferinnen und Ersthelfer in einem Radius von 500 m (Stadtgebiet) bzw. 1000 m (Stadtrandgebiet) aktiviert. Zukünftige Ziele sind die Integration der Standorte von automatischen externen Defibrillatoren (AED) sowie das Erreichen von 1 % der Berliner Bevölkerung zur Teilnahme am System [2].

## Niedrigprioritäre Einsätze

### Abgabe an die kassenärztliche Vereinigung (KV)

Die Verbesserung der Zusammenarbeit der Leitstellen der 112 mit denen des kassenärztlichen Bereitschaftsdiensts mit der Nummer 116117 war zuletzt wiederholt Gegenstand umfassender wissenschaftlicher Untersuchungen [15] und gutachterlicher Empfehlungen an politische Entscheidungsträger [26]. Vor diesem Hintergrund haben das Land Berlin und die KV Berlin bereits 2019 eine Vereinbarung über die Details der Zusammenarbeit in der gemeinsamen Verantwortung für die Notfallnummern 112 und 116117 geschlossen. Inzwischen ist eine ständige elektronische Schnittstelle zwischen den Telefonanlagen und Einsatzleitsystemen etabliert, die die bilaterale, vollständige automatische Weitergabe von Notrufgesprächen einschließlich erhobener Daten und Notrufabfrageergebnisse ohne zusätzlichen Gesprächskontakt der Leitstellen untereinander sicherstellt. Bei Notrufen in der Leitstelle 112, bei denen sich durch die standardisierte Notrufabfrage herausstellt, dass das niedrigprioritäre Hilfeersuchen besser durch das Leistungsangebot der kassenärztlichen Notdienststrukturen als durch jenes des Rettungsdiensts bedient werden kann, erfolgt rund um die Uhr die elektronische Weitergabe per Schnittstelle an die Leitstelle der 116117 und dort zunächst die softwaregestützte, standardisierte Nachtriage mit der bundeseinheitlichen Software SmED [13]. Von täglich durchschnittlich ca. 2300 Hilfeersuchen am Notruf 112 können im Land Berlin zur Zeit nach standardisierter Notrufbefragung bis zu 160 Hilfeersuchen pro Tag an die 116117 zur SmED-Nachtriage weitergegeben werden. Anschließend werden diese Hilfeersuchen, je nach fachlich gebotener Erfordernis, an einen telemedizinischen Beratungsarzt (ca. 25 %), den KV-ärztlichen Hausbesuchsdienst (ca. 25 %), einen privaten Krankentransport (ca. 7 %) oder zur Versorgung in einer vertragsärztlichen Praxis beziehungsweise KV-Notfallpraxis (ca. 40 %) weitergeleitet. Rund 3 % der von der 112 an die 116117 weitergegebenen Hilfeersuchen müssen aufgrund einer in der SmED-Nachtriage festgestellten höheren medizinischen Dringlichkeit oder fehlender anderer Versorgungsmöglichkeiten in den vertragsärztlichen Notfallstrukturen schlussendlich doch mit Einsatzmitteln des Rettungsdiensts beschickt werden.

Von täglich ca. 1000 Hilfeersuchen, die sich im Land Berlin primär an die 116117 wenden, müssen nach SmED-Triage rund 30 aufgrund eines dringlichen Notfalls zu einem Einsatz des Rettungsdiensts heraufgestuft werden. Hierzu wird der entsprechende Datensatz ebenso automatisch per Schnittstelle in das Einsatzleitsystem der 112 übermittelt. Hier erfolgt erneut eine systematische Erfassung mittels des standardisierten Notrufabfrageprotokolls.

Insgesamt kann in einem bereits gut funktionierenden System eines gemeinsamen, virtuellen Notfallleitsystems festgestellt werden, dass jenseits entsprechender Vereinbarungen und technischer Voraussetzungen die standardisierte Notrufabfrage der 112 sowie die softwaregestützte medizinische Ersteinschätzung der 116117 wesentlich zur zielgerichteten und rechtssicheren, leitstellenübergreifenden Zusammenarbeit beitragen. Untersuchungen zu den durch die Berliner Notfallrettung versorgten Patienten zeigten zuletzt außerdem eine Diskrepanz zwischen der am Notfallort durch die Einsatzkräfte vermuteten Erkrankungs- oder Verletzungsschwere und dem in der Notaufnahme angewendeten Manchester Triage System und insgesamt eine hohe Krankheitslast bei den durch den Rettungsdienst versorgten Patienten [18]. Ob durch eine Ausweitung spezifischerer, aufsuchender, notfallmedizinischer Versorgungsangebote, beispielsweise durch multiprofessionelle Teams im Bereich von Psychiatrie, Pflege und Sozialarbeit, das Einsatzaufkommen klassischer, stark belasteter Einsatzmittel insbesondere im Rettungsdienst entlastet werden könnte, sollte im Rahmen von weiteren Modellvorhaben erprobt werden (Tab. 4).

**Table 4:** 

Standardisierte Notrufabfrage	Etablierung einer standardisierten, softwaregestützten Notrufabfrage
	Regelmäßige Evaluation der Dispatch Codes (Code Reviews)
	Systematisches Qualitätsmanagement, ggf. mit Etablierung von QM-Lenkungsausschuss, QM-Ausschuss und QM-Team
	Qualitätssicherung mit Fallauswertungen
	Regelmäßige Aus- und Weiterbildung sowie Feedback- und Fehlerkultur zur Qualitätsverbesserung
Optimierung der Dispositionsstrategie	Ortung der nächstgelegenen Einsatzfahrzeuge und „Nächste-Fahrzeug-Strategie“
	Austauschdisposition
	Etablierung einer systematischen First-Responder-Strategie
	Anbindung von Ersthelfer-Apps mit Integration von AED-Standorten
Zusammenarbeit mit dem ärztlichen Bereitschaftsdienst	Weitergabe niedrigprioritärer Einsätze an die Leitstelle des kassenärztlichen Bereitschaftsdiensts (116117)
	Ständige elektronische Schnittstelle zwischen den Leitstellen mit automatisierter Übertragung standardisierter Datenpakete und Notrufabfrageergebnisse in beide Richtungen
Weiteres	Integration multiprofessioneller Teams (aus den Bereichen Psychiatrie, Pflege, Sozialarbeit) in den Rettungsdienst
	Case Management, bspw. für den Umgang mit Frequent Usern
	Weitergabe niedrigprioritärer Einsätze an alternative Versorgungsformen

### Ausblick – multiprofessionelle Teams in der Notfallrettung

Auch wenn es wenige Daten zur Frage gibt, ob mittels der Anwendung einer standardisierten Notrufabfrage vorausgesagt werden kann, ob Hilfeersuchen mit niedriger Dringlichkeit dahingehend identifiziert werden können, ob sie eine prähospitale Behandlung durch den Rettungsdienst benötigen, so gibt es durchaus Hinweise, dass derartige Systeme zuverlässig sind [14].

Um der Problematik von teilweise undifferenzierten Krankenhauszuweisungen und der fehlenden Versorgungsmöglichkeit vor Ort entgegenzutreten, wurde im Oldenburger Land das Projekt „Gemeindenotfallsanitäter“ initiiert. Hierbei werden speziell geschulte Notfallsanitäter zu ausgewählten Hilfeersuchen disponiert, wobei diesen vor Ort einerseits die Aufgabe der Sichtung, aber auch eine Zuweisungs- und Behandlungskompetenz zukommt [12, 38]. Auch in Berlin wurden im Rahmen der COVID-19-Pandemie NotSan-Erkunder als innovatives Einsatzmittel eingeführt. Hierbei sollte das Konzept in erster Linie dazu dienen, vermeidbare Transporte von Patientinnen und Patienten mit grippaler Symptomatik in die umliegenden Kliniken zu reduzieren [5]. In Niederösterreich wurden als Pilotprojekt sogenannte Acute Community Nurses zur Unterstützung des Rettungsdiensts etabliert. Hierbei geht es in erster Linie darum, adäquat auf den steigenden psychosozialen und pflegerischen Bedarf in Akutsituationen reagieren zu können. Vor allem soll es durch den zusätzlichen Einsatzwert der Acute Community Nurses möglich sein, manche Patienten in der Häuslichkeit zu belassen und Transporte in Kliniken zu vermeiden. Hierfür bietet die standardisierte Notrufabfrage die entsprechende Software „LowCode“, die hier durch die Anbindung an das *Medical Priority Dispatch System* optimale Synergien ergibt, sodass niedrigschwellige Hilfeersuchen unmittelbar an Emergency Community Nurses übergeben werden können, um dort standardisiert weiterbearbeitet zu werden [25].

Es ist derzeit kaum möglich, Patienten mit psychosozialem Hilfeersuchen einer geeigneten Versorgungsstruktur zuzuführen. Beim London Ambulance Service werden Mitarbeiterinnen und Mitarbeiter vermehrt im Umgang mit psychiatrischen Erkrankungen und psychosozialen Krisen geschult, weiterhin werden bereits seit dem Jahr 2015 sog. Mental Health Nurses in der Leitstelle eingesetzt. Auf der Basis erfolgt zunächst eine Risikobewertung im Ergebnis der Notrufabfrage, weiterhin aber auch die Unterstützung bei der Anbindung an eine geeignete Versorgungseinrichtung [11, 20]. Fachkundiges Personal steht somit als Unterstützung für die Disponenten der Leitstelle, aber auch für Einsatzkräfte zur Verfügung und kann beispielsweise telefonisch unterstützen.

Weiterhin wurde im Jahr 2018 ein Einsatzmittel (Mental Health Joint Response Car) eingeführt, welches mit einem Paramedic und einer Mental Health Nurse besetzt ist. Im Ergebnis konnte der Anteil von Patientinnen und Patienten mit psychosozialem Hilfeersuchen, die im Südosten Londons in Krankenhäuser transportiert wurden, von 52 % auf 18 % mehr als halbiert werden [21, 22]. Durch die Arbeit im Team profitieren weiterhin die Paramedics, da die Kenntnisse im Umgang mit psychiatrischen Erkrankungen kontinuierlich verbessert werden. Für das Land Berlin wurde die Idee, sogenannte multiprofessionelle Kriseninterventionsteams als Reaktion auf Notrufe von Menschen, die sich in einem psychischen Ausnahmezustand befinden, an den Rettungsdienst anzubinden, im Koalitionsvertrag für die aktuelle Legislaturperiode festgehalten [32]. Hier bleibt abzuwarten, wie sich die Umsetzung gestalten wird.

### Ausblick – Case Management

Weiterhin bietet die standardisierte Notrufabfrage auch die Möglichkeit, ein Case Management, beispielsweise für den Umgang mit Frequent Usern, unmittelbar in der Leitstelle zu etablieren. In den Rettungsleitstellen des London Ambulance Service wird neben der Funktion des Call Takers und des Dispatchers die Funktion eines Case Managers regelhaft besetzt. Dies bietet die Möglichkeit, dass bei eingehenden Hilfeersuchen mit niedriger Dringlichkeit auf alternative Versorgungshilfen zugegriffen wird. Hier erfolgt auch die Verzahnung des in London bereits etablierten Frequent Caller Case Managements mit eingehenden Notrufen von bekannten Frequent Callern. Nach Identifizierung und Fallbesprechung besteht somit eine Möglichkeit, ein durch die ärztliche Leitung freigegebenes Protokoll (patientenorientierte Handlungsanweisung bzw. patientenorientiertes Protokoll [POP]) im System zu hinterlegen. Somit besteht für die Call Taker die Möglichkeit, bei Anrufen eines vorbekannten Frequent Callers auf dieses spezifisch auf den jeweiligen Frequent Caller zugeschnittene Protokoll zurückzugreifen, sodass eine Beschickung mit einem Einsatzmittel der Notfallrettung vermieden werden kann.

#### Infobox 1 Ablauf der Notrufabfrage in Berlin

- Einstiegsfragen – 1. Notfallcheck
  - „Berliner Feuerwehr, wo genau ist der Notfallort?“
    (Erfragen und Verifizierung von Einsatzadresse und Rückrufnummer)
  - Ggf. unmittelbare erste Disposition bei vermutetem medizinischem Herz-Kreislauf-Stillstand
  - Fragen zu Alter, Geschlecht, Atmungszustand, Erhalt der Bewusstseinslage
- Schlüsselfragen – 2. Notfallcheck
  - (Auswahl eines Hauptbeschwerdeprotokolls)
  - Erfragen zusätzlicher Informationen zum Hilfeersuchen
  - Im Ergebnis differenziertes Zustandsbild
- Priorisierung des Einsatzanlasses und Abbildung in Form eines mehrstelligen Codes
- Erste Hinweise und Anleitung zu Sofortmaßnahmen

#### Infobox 2 Gezielte QM-Fallauswertungen im Notruf-QM

- Danksagungen für die Arbeit am Notruf
- Beschwerden und Haftungsfälle im Kontext der Notrufbearbeitung
- Fälle für das einsatzbezogene QM der ärztlichen Leitung (bspw. eine spezielle Traumareanimation mit hoher Öffentlichkeitswirksamkeit)
- Besondere Codes oder Bearbeitungspfade im Rahmen eines systemischen Fokusfeedbacks (bspw. Code 31D03 Bewusstlosigkeit mit effektiver versus Code 31D01 Bewusstlosigkeit mit ineffektiver Atmung)
- Kalibrierungsfälle, also eine Mehrfachbewertung eines einzigen Falls, zum Abgleich der Bewertungsstandards innerhalb des QM-Teams

## Fazit für die Praxis

- Neben bereits etablierten Elementen ist die Umsetzung weiterer Strategien hin zu einem effizienten gemeinsamen Notfallleitsystem notwendig.
- Die standardisierte, softwaregestützte Notrufabfrage entspricht dem aktuellen Stand der Wissenschaft, eine flächendeckende Einführung ist zur effektiven und rechtssicheren Abwicklung erforderlich.
- Die systematische Umsetzung und Dokumentation einer georeferenzierten, softwaregestützten „Nächstes-Fahrzeug-Strategie“ und einer automatischen Austauschdisposition im Einsatzleitsystem senkt Mortalität und Morbidität von Notfallpatienten im Rettungsdienst, erhöht die Einsatzmittelverfügbarkeit und sorgt im Einklang mit dem Stand der Technik für eine gesteigerte Rechtssicherheit bei Dispositionsentscheidungen.
- Eine intensive Zusammenarbeit zwischen Leitstellen der Notfallrettung und der kassenärztlichen Vereinigungen sorgt für optimierte Ressourcennutzung und Patientenversorgung.
- Die Einführung neuer interprofessioneller Einsatzmittel in der Notfallrettung sowie die Einbindung von Ersthelfern verbessert die bedarfsspezifische Versorgung.
